# TAT‐LBD‐Ngn2‐improved cognitive functions after global cerebral ischemia by enhancing neurogenesis

**DOI:** 10.1002/brb3.2847

**Published:** 2022-12-10

**Authors:** Bin Feng, Sansan Jia, Liya Li, Jiajia Wang, Fang Zhou, Xingchun Gou, Qiang Wang, Lize Xiong, Yi Zeng, Haixing Zhong

**Affiliations:** ^1^ Department of Anesthesiology and perioperative medicine Xijing Hospital, The Fourth Military Medical University Xi'an Shaanxi China; ^2^ Shaanxi Key Laboratory of Brain Disorders & Institute of Basic and Translational Medicine Xi'an Medical University Xi'an China; ^3^ Department of Radiation Oncology Xijing Hospital, The Fourth Military Medical University Xi'an Shaanxi China; ^4^ Department of Anesthesiology The Second Affiliated Hospital of Dalian Medical University Dalian Liaoning China; ^5^ Department of Anesthesiology The First Affiliated Hospital of Xi'an Jiaotong University Xi'an Shaanxi China; ^6^ Department of Anesthesiology and Perioperative Medicine, Shanghai Fourth People's Hospital Tongji University School of Medicine Shanghai China

**Keywords:** global cerebral ischemia, neurogenesis, neurogenin2, post‐stroke neurocognitive disorders, stroke

## Abstract

**Background:**

Stroke is the major cause of adult neurocognitive disorders (NCDs), and presents a significant burden on both of the families and society. To improve the cerebral injury, we generated a blood–brain barrier penetrating peptide TAT‐LBD‐Ngn2, in which Ngn2 (Neurogenin2) is a classical preneural gene that enhances neurogenesis, and neural precursor cells survival and differentiation. We previously demonstrated that it has a short‐term protective effect against cerebral ischemia‐reperfusion injury. However, it is uncertain if TAT‐LBD‐Ngn2 could promote neurogenesis to exhibit long‐term therapeutic impact.

**Methods and results:**

In present study, TAT‐LBD‐Ngn2 was administered for 14 or 28 days following bilateral common carotid arteries occlusion (BCCAO). After confirming that TAT‐LBD‐Ngn2 could cross the brain blood barrier and aggregate in the hippocampus, we conducted open field test, Morris water maze and contextual fear conditioning to examine the long‐term effect of TAT‐LBD‐Ngn2 on cognition. We discovered that TAT‐LBD‐Ngn2 significantly improved the spatial and contextual learning and memory on both days 14 and 28 after BCCAO, while TAT‐LBD‐Ngn2 exhibited anxiolytic effect only on day 14, but had no effect on locomotion. Using western blot and immunofluorescence, TAT‐LBD‐Ngn2 was also shown to promote neurogenesis, as evidenced by increased BrdU^+^ and DCX^+^ neurons in dentate gyrus. Meanwhile, TAT‐LBD‐Ngn2 elevated the expression of brain derived neurotrophic factor rather than nerve growth factor compared to the control group.

**Conclusions:**

Our findings revealed that TAT‐LBD‐Ngn2 could dramatically promote learning and memory in long term by facilitating neurogenesis in the hippocampus after global cerebral ischemia, indicating that TAT‐LBD‐Ngn2 may be an appealing candidate for treating poststroke NCD.

## Graphical abstract

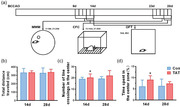

## 1 INTRODUCTION

With medical advances, approximately 25.7 million patients survived from stroke each year, causing 113 million disability‐adjusted life years (DALYs) (Feigin et al., 2015) . In these stroke survivors, poststroke neurocognitive disorder (NCD) is a major outcome event, and associated with mortality, disability, recurrence of major vascular events, and medical expenses. According to the most recent meta‐analysis, the overprevalence of poststroke NCD is up to 53.4% (Barbayet al., 2018). Furthermore, the major poststroke NCD (i.e., vascular dementia), which accounts for 1/5–1/3 of NCD (Barbay et al., 2018; Yang et al., 2020), constitutes a heavy health burden to both of the family and society. As one of the most important social medical challenge, to promote cognitive rehabilitation after stroke, will not only improve the quality of life for patients, but also decline the associated medical cost. However, no established treatments for poststroke NCD is available so far.

Though there remains controversial, neurogenesis in hippocampus is believed to improve learning and memory (Almulla et al., 2021), which has been proven in Alzheimer's disease studies (Moreno‐Jimenez et al., 2019; Richetin et al., 2020). Therefore, poststroke NCD patients (i.e., vascular dementia) may also benefit from neurogenesis as seen in AD (Koh et al., 2017). Neurogenin 2 (Ngn2), which belongs to the basic helix‐loop‐helix (bHLH) family, is one of the most important and extensively studied molecules that regulate neurogenesis and neuronal development. Actually, Ngn2 could initiate neurogenesis, and regulate cell fate commitment, neuron migration, axon guidance, and cell maturation (Hand et al., 2011; Heng et al., 2008; Schörniget al., 2021; Serreet al., 2012). Some researches have verified that Ngn2 was sufficient to stimulate embryonic stem cells (Thoma et al., 2012), astrocytes, oligodendrocytes precursor cells (Blum et al., 2011), and skin‐derived precursors (Dai et al., 2015) differentiation into mature neurons. Furthermore, Ngn2 promoted the translated neural precursor cells or stem cells to survive, differentiate, migrate, and mature (Ho et al., 2016; Ishikawa et al., 2020; Tang et al., 2016). Therefore, Ngn2 may be an ideal candidate to promote neurogenesis for poststroke NCD therapy. However, the Ngn2 is too large to penetrate the blood–brain barrier (BBB).

In the previous study, we created the TAT‐LBD‐Ngn2 peptide by fusing Ngn2 with human immunodeficiency virus (HIV) trans‐activator of transcription (TAT) and Laminin binding domain (LBD) to achieve selectively aggregation in the ischemic area in the brain (Roll et al., 2014). We confirmed that the TAT‐LBD‐Ngn2 could attenuate cerebral ischemia‐reperfusion injury by inhibiting neuronal degeneration and apoptosis, and improve short‐term cognition by regulating caspase‐dependent and mitochondrial apoptotic pathways (Deng et al., 2013; Zhao et al., 2018).

Here, we focused on the long‐term effect of TAT‐LBD‐Ngn2 on neurological functional recovery for 14 and 28 days after global cerebral ischemia (GCI). Using neurobehavioral tests, immunohistochemistry, and western blot, we found that TAT‐LBD‐Ngn2 significantly improved both spatial and contextual learning and memory impairments in a long term by promoting adult neurogenesis, and increasing the expression of brain derived neurotrophic factor (BDNF) but not nerve growth factor (NGF).

## 2 MATERRIALS AND METHODS

### 2.1 Animals and experimental design

All procedures were performed according to the Guidelines for Animal Experimentation as approved by the Ethics Committee for Animal Experimentation of the Fourth Military Medical University, Xi'an, China. Seventy adult SPF C57BL/6 mice, weighing 22–25 g, were provided by Experimental Animal Center of the Fourth Military Medical University. All animals were housed in group of 4 under a 12:12 h light/dark cycle in temperature‐controlled environment (22–24°C) with food and water available ad libitum.

For all experiments, mice were subjected to transient GCI, and then randomly assigned to TAT‐LBD‐Ngn2 (TAT) group for 14 days, TAT group for 28 days, Control (Con) group for 14 days, or Con group for 28 days. In the TAT‐LBD‐Ngn2 (TAT) group, 250 μg/kg/day of TAT‐LBD‐Ngn2 (Deng et al., 2013) was administered intraperitoneally, while equal volume of 0.1 M phosphate buffered saline (PBS) solution (pH 7.4) was injected in Control (Con) group for 14 or 28 days. For neurogenesis analysis, 5‐bromo‐2‐deoxyuridine (BrdU, 50 mg/kg, dissolved in 0.9% sterile NaCl, Sigma‐Aldrich, USA) was given intraperitoneally twice daily for 7 days after surgery, and repeated at the third week to reduce its toxicity and improve its efficiency. Before day 14 or 28 after GCI, the behavioral tests were performed. Afterward, the mice were sacrificed for immunofluorescence and western blot studies.

### 2.2 Transient global forebrain ischemia model

Under 2% isoflurane anesthesia (delivered by 1 L/min 100% oxygen), mice (*n* = 3 for each group) were shaved and sterilized. A probe of laser Doppler flow meter (Perimed, Stockholm, Sweden) was fixed on the exposed skull (A‐P: −1.7 mm, M‐L: +2 mm) by dental cement. Hereafter, mice were shifted to supine position carefully to gently blunt dissected and clipped the bilateral common carotid arteries for 20 min with continuous regional cerebral blood flow (rCBF) monitoring. The mice were involved in the further study, unless the rCBF reduced more than 70% of baseline, suggesting bilateral common carotid arteries occlusion (BCCAO) model was established successfully (Kitagawa et al., 1998). A heated surgical plate was used throughout the surgical procedure to keep the animals’ temperature.

### 2.3 Neurobehavioral tests

After habituated for 3 days, mice (*n* = 10 for each group) were assigned to Morris water maze (MWM), contextual fear conditioning (CFC), and open field test (OFT), respectively (Figure 2a).

#### 2.3.1 Open‐field test

The OFT was conducted on day 14 or 28 as described previously (Dong et al., 2020; Sun et al., 2021). After gently placed into the center zone of the open field chamber (40 × 40 × 40 cm), mice (*n* = 10 for each group) were allowed to move freely under dim light for 5 min after a 2‐min habituation period. Total distance travelled, the line crossings in the center, and the time spent in the center were collected and analyzed automatically (Topscan, Clever Sys Inc., Reston, VA, USA). Before each test, the chamber was cleaned and deodorized with 70% ethanol.

#### 2.3.2 Morris water maze test

The MWM was performed from day 9 or 23 after GCI for 5 consecutive days. Facing the wall, mice (*n* = 10 for each group) were gently released into the white‐colored stainless‐steel circular tank (122 cm in diameter, with a hidden platform 1 cm below the water and paper clues on each quadrant wall) in a quiet and indirect illuminated room. Animal was allowed to explore the hidden platform for 60 s. If it failed, it would be led to the platform and kept for 15 s. Every day, mice were tested from four different quadrants with an interval of 30 min. Twenty‐four hours after last training, mice were released into the tank without platform for 30 s to assess the reference memory. The latency to reach the goal, the time spent in target quadrant, and the number of platform‐site crossovers were monitored and analyzed by an automatic tracking system (Topscan, Clever Sys Inc.).

#### 2.3.3 Contextual fear conditioning

The CFC was conducted from day 13 or 27 after GCI. The mice (*n* = 10 for each group) were placed into the chamber individually for 5 min to habituate. And then, three times of foot shocks were given (0.7 mA, 2 s with 35–60 s interval), followed by another 1 min kept in the chamber. Twenty‐four hours later, mice were moved to the same context for 5 min to test the contextual memory retrieval. Freezing behavior was defined as no movement for at least 2 s by automatically recorded and analyzed (Version 2.26, Actimetrics Inc., USA). Before each test, the conditioning chamber was cleaned and deodorized with 70% ethanol.

### 2.4 Tissue preparations and immunofluorescence

Under pentobarbital anesthesia, mice (*n* = 3 for each group) were perfused transcardially with 0.1 M PBS and 4% cold paraformaldehyde successively. The brains were harvested and dehydrated by 20% and 30% sucrose (dissolved in PBS) consecutively. Brains were sliced coronally (12 μm thickness) by cryostat microtome (Leica CM1900, Germany)

To examine BrdU expression, sections were rinsed, incubated in 2 M HCl at 37°C for 30 min, and treated with 0.1 M borate buffer (PH 8.5) for 10 min successively. After being washed, the slices were incubated in 10% goat serum at 37°C for 60 min, followed by mouse monoclonal anti‐BrdU (1:400, Sigma‐Aldrich, USA) and guinea pig anti‐NeuN (1:500, Synaptic system, Germany) overnight at 4°C. Afterward, secondary antibody mixture (donkey anti‐mouse Alexa Fluor 594, 1:500, Abcam, USA; donkey anti‐guinea pig FITC, 1:500, Burlingameor, USA) was applied to rinsed sections for 2 h at room temperature (RT).

For other immunofluorescence detection, the sections were treated with rabbit anti‐DCX (1:200, Abcam, USA), rabbit anti‐BDNF (1:200, Abcam, UK), rabbit anti‐NGF (1:100, GeneTex, USA), or anti‐(His)6 mouse monoclonal (1:1000, Abcam, UK) antibody along with guinea pig anti‐NeuN antibody overnight at 4°C, respectively. These slices were rinsed, incubated in secondary antibody solution (donkey anti‐rabbit Alexa Fluor 594, 1:500, Abcam, USA; donkey antiguinea pig FITC, 1:500, Burlingameor, USA) for 2 h at RT. Images were acquired by the confocal microscope (FV1200, Olympus, Japan).

All the BrdU‐immunoreactive (BrdU^+^) and DCX‐immunoreactive (DCX^+^) neurons in the dentate gyrus (DG) were observed and counted from each section (*n* = 3 per group) by a blinded investigator (Image‐Pro Plus version 6.0, Media Cybernetics, USA) (Brown et al., 2003; Maysami et al., 2008).

### 2.5 Western blot

Hippocampus tissues (*n* = 3 per group) were collected and lyzed by RIPA protein extraction buffer containing 1 × protease inhibitor. The total protein concentration was examined by BCA protein assay kit (Pierce, USA). Protein was separated by 10% SDS‐polyacrylamide gels and transferred to polyvinylidene fluoride membrane (Millipore, USA). Then the membranes were incubated within 5% nonfat milk for 1 h at RT, and then rabbit anti‐BDNF (1:1000, Abcam, UK) or rabbit anti‐NGF (1:1000, GeneTex, USA) antibody overnight at 4°C. Rabbit anti‐β‐Tubulin (1:5000, Abcam, UK) was applied as internal standard. The goat antirabbit IgG (Immunoglobulin G) conjugated with horseradish peroxidase (1:10,000; ab205719; Abcam; UK) was added to the membranes for 1 h at RT. At last, the blots were visualized and analyzed by Image J software (Bethesda, USA).

### 2.6 Statistical analysis

All data were presented as mean ± SD and analyzed by GraphPad Prism software6.0 (GraphPad, USA). All the bar figures were analyzed by two‐way ANOVA, while other data were analyzed by two‐way ANOVA‐repeated measurement. Post‐hoc Bonferroni multiple comparison tests was used. Statistical significance was set at *p* < .05.

## 3 RESULTS

### 3.1 TAT‐LBD‐Ngn2 was concentrated in injury regions after GCI

**FIGURE 1:**
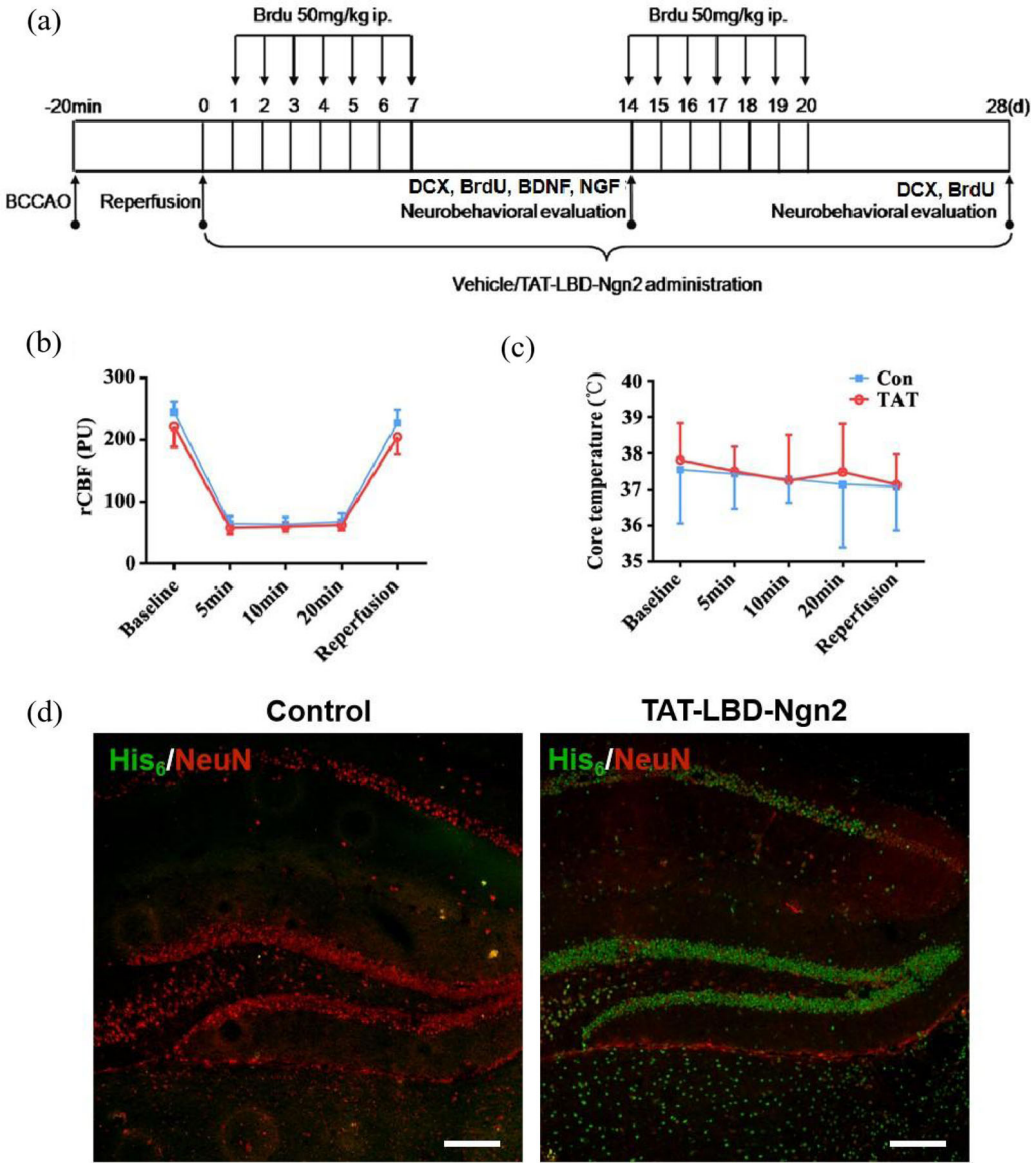
TAT‐LBD‐Ngn2 could gather in the hippocampus after GCI. (a) The schematic diagram of the experimental procedure. (b and c) There were no significant differences in rCBF (b) and core temperature (c) during the BCCAO surgery. (d) The 6‐his marked cells were specifically exhibited in the TAT group but not Con group in the hippocampus. Con, Control; TAT, TAT‐LBD‐Ngn2. Scale bars: 100 μm

The flow diagram of experiments was shown (see Figure 1a). In the GCI model, continuous intraperitoneal injection of Brdu (50 mg/kg) was performed daily on days 1–7 and 14–20, and immunofluorescence staining and behavioral evaluation were performed on days 14 and 28, respectively. The establishment of the GCI model was confirmed, and there were no significant differences in rCBF and body temperature between the Con and TAT groups during the surgery (Figure 1b, c).

The expression of TAT‐LBD‐Ngn2 was investigated by its tag His_6_. We found that the His_6_‐positive cells were concentrated in the hippocampus after 3 days administration of TAT‐LBD‐Ngn2, especially in the DG (Figure 1d), which is one of the most vulnerable regions for ischemia. The results confirmed that TAT‐LBD‐Ngn2 could transport across the BBB to penetrate into neural cells, and selectively gather in the injury regions.

### 3.2 TAT‐LBD‐Ngn2 alleviated long‐term cognitive impairment after GCI

**FIGURE 2:**
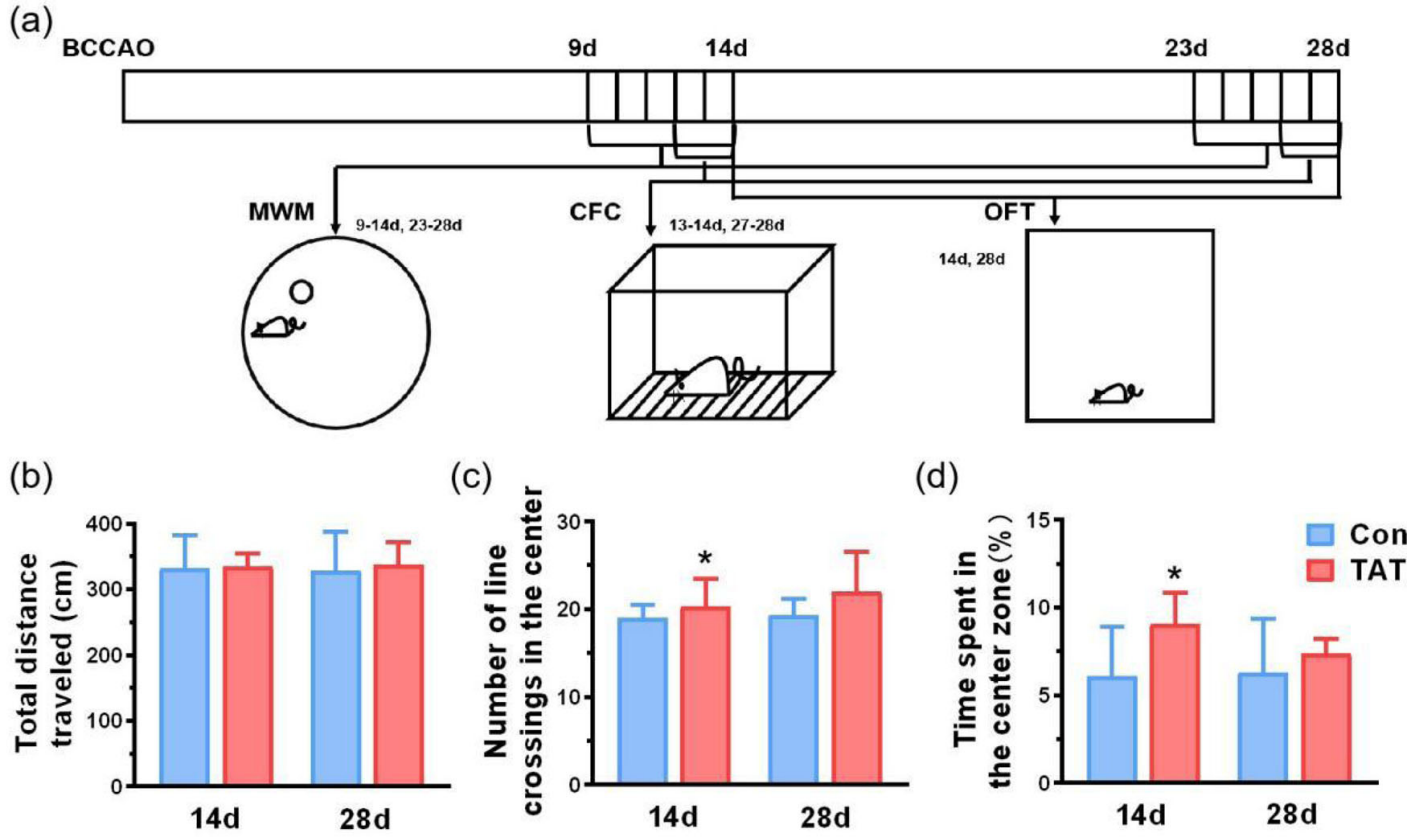
TAT‐LBD‐Ngn2 alleviated anxiety on day 14 after GCI, without affecting locomotion in open field test. (a) The schematic diagram of behavioral tests. (b) The total distance traveled in OFT between Con and TAT groups on days 14 and 28, and there were no significant differences between two groups (*F*(1,34) = 0.1487, *p* = .702). (c) The numbers of line crossing in the center significantly increased in the TAT groups compared with the Con group on day 14 (*F*(1,34) = 4.151, *p* = .0495), but there were no significant differences between two groups (*F*(1,34) = 0.1287, *p* = .902). (d) The time percentage spent in the center zone significantly increased in the TAT groups than the Con group on day 14 (*F*(1,34) = 7.062, *p* = .0119), but there were no significant differences between two groups (*F*(1,34) = 0.2187, *p* = .612). Data are represented as the mean ± SD for each group (*n* = 11 independent samples). For (b–d), significance was determined by two‐way ANOVA followed by post‐hoc Bonferroni multiple comparison analysis. *p < .05

To investigate the long‐term cognitive functions after GCI, a battery of behavioral tests was conducted respectively (Figure 2a).

In the OFT, we found that the locomotor of mice had no significant differences between two groups (*F*(1,34) = 0.1487, *p* = .7022, Figure 2b). Meanwhile, both the number of line crossings in the center (*F*(1,34) = 4.151, *p* = .0495, Figure 2c) and the percentage of time spent in the center zone (*F*(1,34) = 7.062, *p* = .0119, Figure 2d) significantly increase in the TAT groups compared with the control group, specifically on day 14, indicating that TAT‐LBD‐Ngn2 may attenuate anxiety induced by GCI.

**FIGURE 3:**
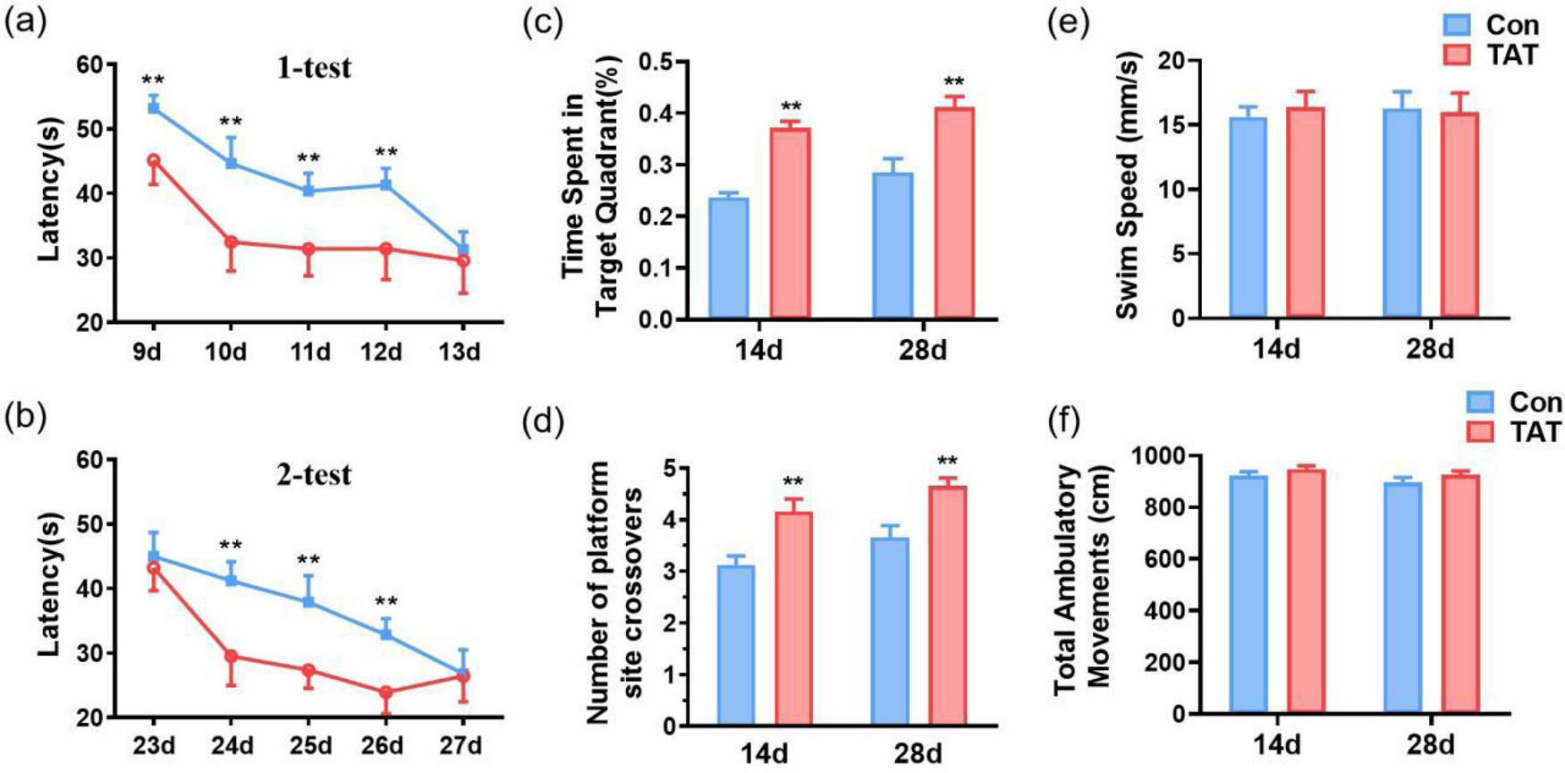
TAT‐LBD‐Ngn2 promoted learning and memory in the Morris water maze. (a) Mice in the TAT group exhibited reduced latency to locate the hidden platform on days 9–13 compared with the Con group (*F*(1, 80) = 105.0, *p* < .0001). (b) Mice in the TAT group exhibited reduced latency to locate the hidden platform on days 23–27 compared than the Con group (*F*(1, 80) = 77.97, *p* < .0001). (c) One day later, mice treated by TAT showed more time spent in the target quadrant than Con group on day 14 and day 28 (*F*(1, 34) = 45.75, *p* < .0001). (d) One day later, mice in the TAT group expressed better retrieval memory than in the Con group, showing as in the numbers of platform site crossover on day 14 and day 28 (*F*(1, 34) = 25.01, *p* < .0001). (e) Swim speed was shown between Con and TAT groups on day 14 and day 28, but there were no significant differences between two groups (*F*(1, 34) = 0.03063, *p* = .8621). (f) Total ambulatory movements were no significant differences between Con and TAT groups on day 14 and day 28 (*F*(1, 34) = 2.87, *p* = .0990). Data are represented as the mean ± SD for each group (*n* = 9 independent samples). For (c–f), significance was determined by two‐way ANOVA followed by post hoc Bonferroni multiple comparison analysis. **p < .001

To examine the spatial learning and memory, MWM was conducted. In the acquisition sessions, the latency to locate the hidden platform in the TAT group was much shorter than that in the Con group during the first (*F*(1, 80) = 105.0, *p* < .0001, Figure 3a) and second tests (*F*(1, 80) = 77.97, *p* < .0001, Figure 3b), especially on the 2nd–4th training days. Twenty four hours later, mice in the TAT group expressed better retrieval memory than in the Con group, showing as more time spent in the target quadrant (*F*(1,34) = 45.75, *p* < .0001, Figure 3c) and platform‐site crossovers (*F*(1,34) = 25.01, *p* < .0001, Figure 3d) on both days 14 and 28. Meanwhile, there were no statistic differences in swim speed (*F*(1,34) = 0.03063, *p* = .8621, Figure 3e) or total ambulatory movements (*F*(1,34) = 2.87, *p* = .0990, Figure 3f).

**FIGURE 4:**
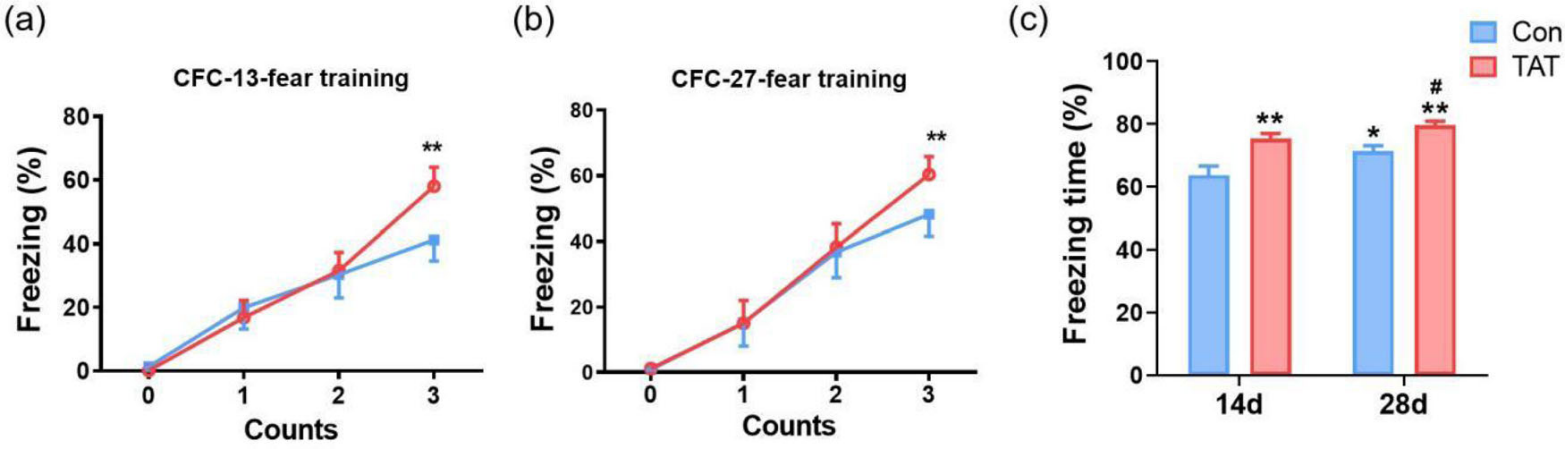
TAT‐LBD‐Ngn2 improved learning and memory in the contextual fear conditioning (CFC). (a) TAT‐LBD‐Ngn2 significantly elevated the freezing time in CFC, especially in the third trail on day 13 (*F*(1, 80) = 8.673, *p* = .0042). (b) TAT‐LBD‐Ngn2 significantly elevated the freezing time in CFC, especially in the third trail on day 27 (*F*(1, 80) = 7.169, *p* = .0090). (c) One day later, mice in the TAT group spent significantly more time in freezing than those in the control group on both days 14 and 28 (*F*(1, 38) = 22.17, *p* < .0001). Data are represented as the mean ± SD for each group (*n* = 9 independent samples). For (a–c), significance was determined by two‐way ANOVA followed by post‐hoc Bonferroni multiple comparison analysis. **p < .01

To further observe the hippocampus‐dependent contextual learning and memory, CFC was performed. Before foot shocks, there were no differences in freezing time between Con and TAT groups. During conditioning, the freezing time progressively increased after each foot shock, indicating that the acquisition sessions were performed successfully. Furthermore, the mice in the TAT group learned faster than those in the Con group on both day 13 (*F*(1, 80) = 8.673, *p* = .0042, Figure 4a) and day 27 (*F*(1, 80) = 7.169, *p* = .0090, Figure 4b), especially on the last trial. When returned to the contextual chamber 1 d after conditioning, mice in the TAT group spent significantly more time in freezing than those in the control group on both days 14 and 28 (*F*(1, 38) = 22.17, *p* < .0001, Figure 4c).

### 3.3 TAT‐LBD‐Ngn2 promoted neurogenesis in DG after GCI

**FIGURE 5:**
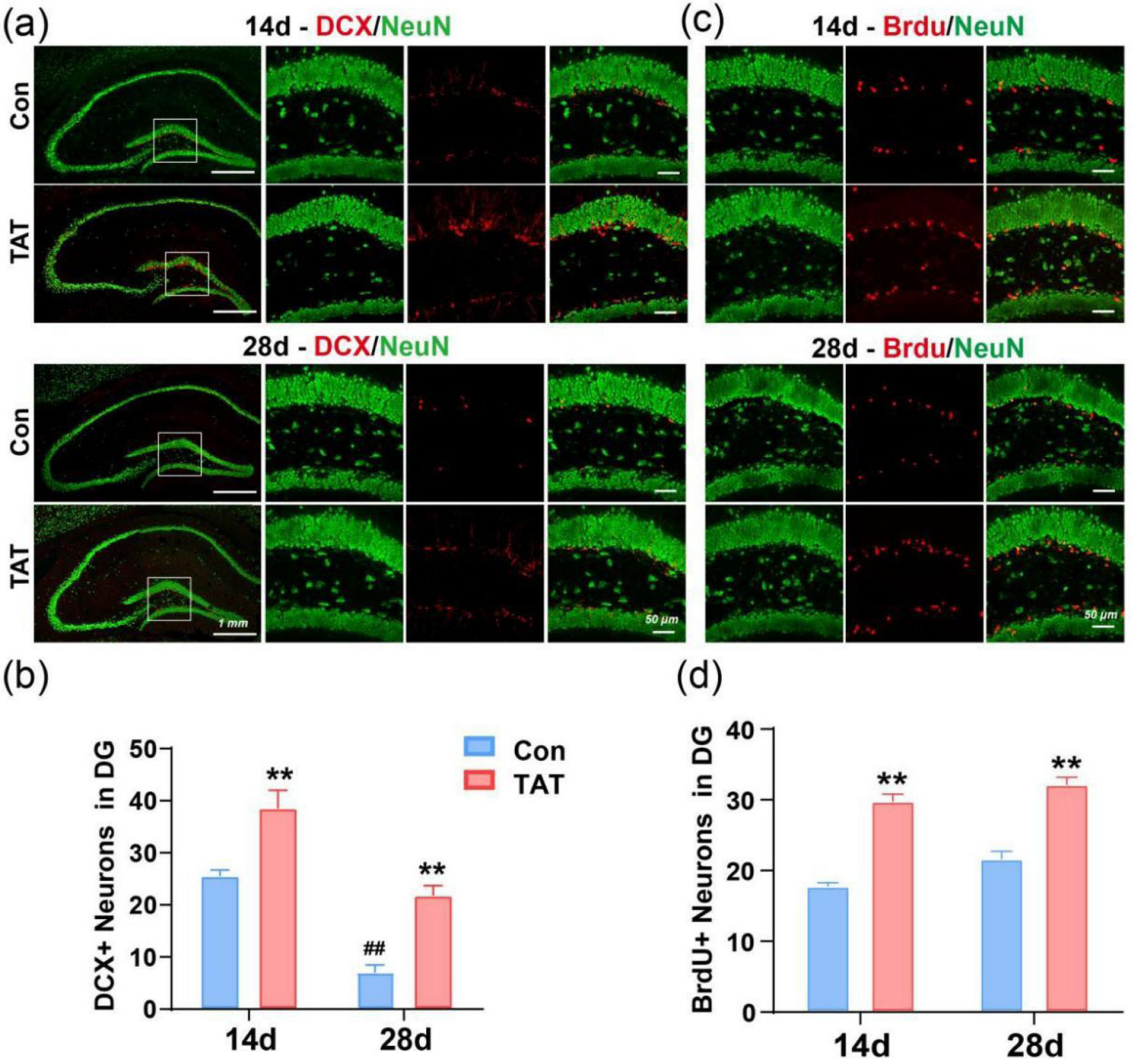
TAT‐LBD‐Ngn2 promoted neurogenesis after GCI. (a) The DCX^+^ and NeuN^+^ cells in the DG on day 14 (up) and day 28 (down) after BCCAO. Scale bar: 50 μm. (b) TAT‐LBD‐Ngn2 increased the number of DCX^+^ cells on both day 14 and day 28 (*F*(1, 12) = 39.03, *p* < .0001) (*n* = 3 independent samples). (c) The BrdU^+^ and NeuN^+^ neurons in the DG on day 14 (up) and day 28 (down) after BCCAO. Scale bar: 50 μm. (d) TAT‐LBD‐Ngn2 increased the number of BrdU^+^ neurons on both day 14 and day 28 (*F*(1, 12) = 136.9, *p* < .0001) (*n* = 3 independent samples). Data are represented as the mean ± SD for each group. For (b, d), significance was determined by two‐way ANOVA followed by post‐hoc Bonferroni multiple comparison analysis. ##*p* < .01; **p < .01

To verify whether the promotion of learning and memory in the TAT‐LBD‐Ngn2 group due to neurogenesis effect of Ngn2, we analyzed the number of DCX^+^ and BrdU^+^ neurons (Couillard‐Despres et al., 2005; Fuss et al., 2010). As expected, the DCX^+^ neurons in DG increased approximately 0.5‐fold on day 14 (*p* = .0082) and 2‐fold on day 28 (*p* = .0031) in the TAT group compared to the Con group (*F* (1, 12) = 39.03, *p* < .0001, Figure 5a, b). The number of BrdU^+^ neurons also elevated in the TAT group compared to the Con group (*F* (1, 12) = 136.9, *p* < .0001, Figure 5c, d), on both day 14 (*p* < .0001) and day 28 (*p* < .0001). All of the above results indicated that TAT‐LBD‐Ngn2 may improve neurogenesis in DG, and maintain the newborn neurons survival.

### 3.4 TAT‐LBD‐Ngn2 increased the expression of BDNF rather than NGF

We also investigated the expression and distribution of BDNF and NGF to further reveal the possible effect of TAT‐LBD‐Ngn2 on neurogenesis.

**FIGURE 6:**
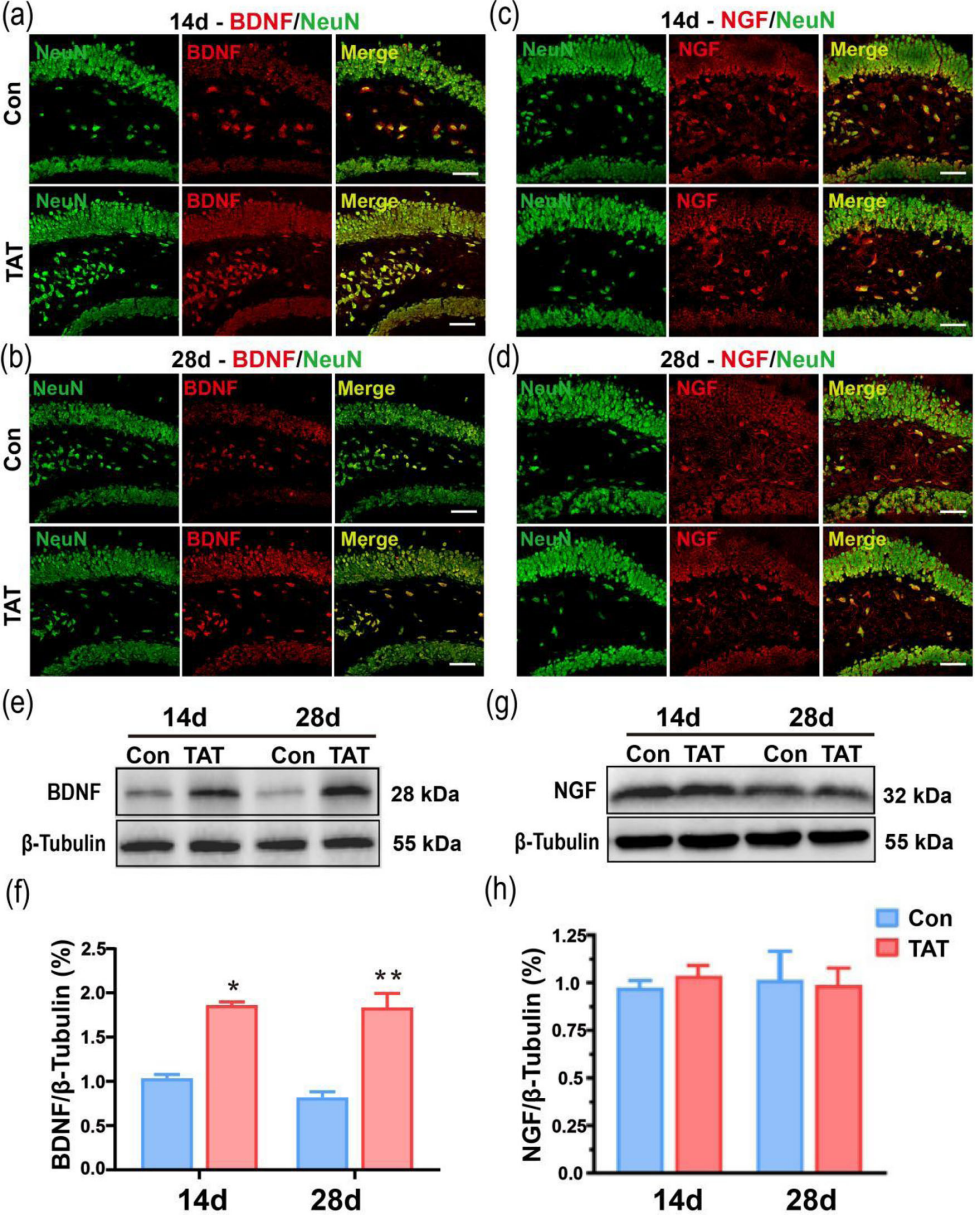
Fourteen and 28 days after ischemia, TAT‐LBD‐Ngn2 increased the expression of BDNF, but not NGF. (a) The distribution of BDNF was, respectively, observed between con and TAT groups on day 14 in the hippocampus. (b) The distribution of BDNF was, respectively, observed between two groups on day 28 in the hippocampus. (c) The distribution of NGF was, respectively, shown in the hippocampus between con and TAT groups on days 14. (d) The distribution of NGF was, respectively, shown in the hippocampus between two groups on day 28. Scale bar: 50 μm. (e, f) Western blot investigation verified the elevation of BDNF in the TAT group in hippocampus than the Con group on both days 14 and 28 (*F*(1, 8) = 32.32, *p* = .0005) (*n* = 3 independent samples). (g, h) Western blot reveals NGF expression no difference between con and TAT groups on days 14 and 28 in the hippocampus (*F*(1, 8) = 0.1042, *p* = .7551) (*n* = 3 independent samples). Data are represented as the mean ± SD for each group. For (f, h), significance was determined by two‐way ANOVA followed by post‐hoc Bonferroni multiple comparison analysis. *p < .05, **p < .01

The immunofluorescence results shown that the expression of BDNF enhanced after 14 and 28 days of TAT‐LBD‐Ngn2 treatment in hippocampus (Figure 6a, b). Western blot investigation further verified the elevation of BDNF in hippocampus on both days 14 and 28 (*F*(1, 8) = 32.32, *p* = .0005, Figure 6e, f). However, the distribution of NGF was not regulated by TAT‐LBD‐Ngn2 in hippocampus on both days 14 and 28 Figure 6c, d). The protein expression of NGF had no significant differences between Con group and TAT group in hippocampus on both days 14 and 28 (*F*(1, 8) = 0.1042, *p* = .7551, Figure 6 g, h). Based on above results, we considered that TAT‐LBD‐Ngn2 may promote neurogenesis associated with BDNF but not NGF in the hippocampus.

## 4 DISCUSSION

Using immunofluorescence staining technique and a battery of neurobehavioral tests, we verified that TAT‐LBD‐Ngn2 could penetrate the BBB and concentrate at hippocampus to attenuate learning and memory impairments after GCI models in long term, up to 28 days after GCI. The anxiety level was also decreased on day 14 but not day 28 after TAT‐LBD‐Ngn2 treatment. Furthermore, the underlying mechanisms may be related to adult neurogenesis, as TAT‐LBD‐Ngn2 treatment increased DCX^+^ and BrdU^+^ neurons persistently in the hippocampus. Besides, the expression of BDNF was upregulated in the hippocampus by TAT‐LBD‐Ngn2, while the expression of NGF was not influenced.

In the previous study, we have created fusion protein TAT‐LBD‐Ngn2, and found that it exhibited neuroprotective effect in short term by inhibiting neuronal degeneration and apoptosis after focal cerebral ischemia, and alleviating caspase‐dependent and mitochondrial apoptotic pathway through BDNF‐TrkB signaling after GCI (Deng et al., 2013; Zhao et al., 2018). However, there is still a gap in the long‐term neuroprotective effect of TAT‐LBD‐Ngn2, which may be more important to benefit patients and society in burden alleviation, because there will be 65.7 million people with major NCD in 2030 (Silva et al., 2018). In consistent with the previous study, we utilized the BCCAO model, which leads to more NCD instead of motor deficiency (Zhao et al., 2018). Furthermore, we mainly concentrated on the hippocampus based on three reasons. First, previous study has confirmed that the expression of Ngn2 decreased mainly in the hippocampus after GCI (Zhao et al., 2018). Second, the GCI model has been proven to induce neuronal damages mainly in the hippocampus and the cortex rather than significant infarction (León‐Moreno et al., 2020). Last but not least, we mainly investigated the neurogenesis, which occurs in the DG of the hippocampus rather than the cortex. As we expected, long‐term application of TAT‐LBD‐Ngn2 could improve learning and memory after stroke, by promoting adult neurogenesis in the DG. These results further support that the neurogenesis is helpful in restoring cognitive impairments. Considering that we did not observe any side effect after long‐term drug administration, the fusion protein may be an ideal candidate for stroke therapy. Besides, the TAT‐LBD‐Ngn2 may produce extra cognitive protective effect by facilitating injury neurons survived.

Anxiety is another persistent symptom in out‐of‐hospital GCI patients (Boyce et al., 2019). In animal studies, anxiety behavior could persist 14 days in elevated plus‐maze and 28 days in OFT after 17 min GCI without rCBF monitor (Soares et al., 2013), while anxiety levels went normal on days 15 and 30 after 10 min BCCAO (Milot et al., 2009). Our results shown that TAT‐LBD‐Ngn2 exhibited an anxiolytic effect after 20 min GCI on day 14 rather than on day 28, confirming that TAT‐LBD‐Ngn2 could attenuate anxiety. The negative anxiolytic effect on day 28 may be on account of disappeared anxiety, or different extent and duration of BCCAO, which may lead to different injury brain regions (Shenet al., 2011; Soares et al., 2013; Yoshioka et al., 2011).

It has been reported the GCI may lead to learning and memory impairments up to 125 days until the CA1 neurons reappeared (Aoyagi et al., 1998). During the repairing process, hippocampal neurogenesis peaks at 7–10 days, and then go down to the baseline within 2–4 weeks (Kawai et al., 2004). Other research also found that the DCX^+^ neurons decreased in the DG 14 and 28 days after GCI (Pforte et al., 2005; Soares et al., 2013). These data are consistent with our results that the DCX^+^ and BrdU^+^ neurons were less on day 28 than those on day 14 in the control group. On the other hand, 50% of BrdU^+^ neurons eliminated from the adult DG within 22‐day period because of apoptosis (Dayer et al., 2003). However, we found that the DCX^+^ and BrdU^+^ neurons on day 28 were still as many as on the day 14 in the TAT‐LBD‐Ngn2 group. There may be three reasons contributed to the increased new born neurons. First, the TAT‐LBD‐Ngn2 may persist facilitating neurogenesis and keep it at a relatively high level for at least 28 days, because Ngn2 acts as a classical proneural gene and is responsible for regulating the birth of early‐born neurons (Aslanpouret al., 2020). Second, TAT‐LBD‐Ngn2 inhibited the new born neurons apoptosis, as our previous study confirmed that the drug could inhibit neuronal degeneration and apoptosis, and enhance cognitive functional recovery in the acute stage after focal cerebral ischemia (Zhao et al., 2018). Last but not least, TAT‐LBD‐Ngn2 may induce survival‐promoting neurotrophic factors, hormones, and other extracellular signals expression by the survival neural cells, which, in turn, promoted the neurogenesis (Kuhn et al., 2015). Our results also shown that the TAT‐LBD‐Ngn2 upregulated the expression of BDNF, which played a crucial role in stimulating the differentiation, proliferation, and survival of newly generated neurons by interference with apoptosis‐inducing signaling pathways (Rossi et al., 2006). Kurozumi et al. also indicated that transplantation of BDNF overexpression mesenchymal stem cells (MSCs) could improve neurological functional recovery by reducing apoptotic neural cells after transient cerebral ischemia (Kurozumiet al., 2004).

There are some limitations. At first, we only investigate the neurogenesis, but did not further clarify their differentiation, maturation, migration, the apoptosis, or integration of the new born neurons to further confirm their fate and functions. Second, we found that the BDNF was increased by TAT‐LBD‐Ngn2, and proposed that the elevation was attributed to the newborn neurons. However, the BDNF was also enhanced in the acute phase after GCI; therefore, the further reason and effects need to be revealed in the following research.

## 5 CONCLUSION

In summary, we have demonstrated for the first time that long‐term administration of TAT‐LBD‐Ngn2 could significantly improve learning and memory, and reduce anxiety after GCI. The underlying mechanisms may be TAT‐LBD‐Ngn2 crossed the BBB, aggregated at the lesion regions, and promoted neurogenesis partly by increasing the expression of BDNF. Our serial studies may shed light on the rescue of cognitive impairment after stroke.

## AUTHOR CONTRIBUTIONS

HXZ and YZ conceived and designed the study. BF and SSJ performed the experiments, collected, and analyzed the data, and wrote the manuscript. LYL, JJW, and FZ collected and analyzed the data. XCG, QW, and LZX provided resources and concepts. All the authors agreed for submission and approved the final version of the manuscript accepted for publication.

## CONFLICT OF INTEREST

The authors claim that there are no conflicts of interest.

### PEER REVIEW

The peer review history for this article is available at https://publons.com/publon/10.1002/brb3.2847

## Data Availability

The data used to support the findings of this study are available from the corresponding author upon request.

## References

[brb32847-bib-0001] Almulla, A. Y. H. , Mogulkoc, R. , Baltaci, A. K. , & Dasdelen, D. (2021). Learning, neurogenesis, and effects of flavonoids on learning. Mini ‐ Reviews in Medicinal Chemistry, 7, 10.2174/1389557521666210707120719 34238155

[brb32847-bib-0002] Aoyagi, A. , Saito, H. , Abe, K. , & Nishiyama, N. (1998). Early impairment and late recovery of synaptic transmission in the rat dentate gyrus following transient forebrain ischemia in vivo. Brain Research, 799(1), 130–137. 10.1016/S0006-8993(98)00465-X 9666102

[brb32847-bib-0003] Aslanpour, S. , Han, S. , Schuurmans, C. , & Kurrasch, D. M. (2020). Neurog2 acts as a classical proneural gene in the ventromedial hypothalamus and is required for the early phase of neurogenesis. Journal of Neuroscience, 40(18), 3549–3563. 10.1523/JNEUROSCI 2610–19. 2020 32273485 PMC7189762

[brb32847-bib-0004] Barbay, M. , Diouf, M. , Roussel, M. , & Godefroy, O. (2018). Systematic review and meta‐analysis of prevalence in post‐stroke neurocognitive disorders in hospital‐based studies. Dementia and Geriatric Cognitive Disorders, 46, 322–334. 10.1159/000492920 30504699

[brb32847-bib-0005] Blum, R. , Heinrich, C. , Sánchez, R. , Lepier, A. , Gundefinger, E. D. , Berninger, B. , & Götz, M. (2011). Neuronal network formation from reprogrammed early postnatal rat cortical glial cells. Cerebral Cortex, 21(2), 413–424. 10.1093/cercor/bhq107 20562320

[brb32847-bib-0006] Boyce, L. W. , Goossens, P. H. , Moulaert, V. R. , Pound, G. , & van Heugten, C. M. (2019). Out‐of‐hospital cardiac arrest survivors need both cardiological and neurological rehabilitation! Current Opinion in Critical Care, 25(3), 240–243. 10.1097/MCC.0000000000000609 31022086

[brb32847-bib-0007] Brown, J. , Cooper‐Kuhn, C. M. , Kempermann, G. , Praag, H. V. , Winkler, J. , Gage, F. H. , & Kuhn, H. G. (2003). Enriched environment and physical activity stimulate hippocampal but not olfactory bulb neurogenesis. European Journal of Neuroscience, 17(10), 2042–2046. 10.1046/j.1460-9568.2003.02647.x 12786970

[brb32847-bib-0008] Couillard‐Despres, S. , Schaubeck, S. , Aigner, R. , Vroemen, M. , Weidner, N. , Bogdahn, U. , Winkler, J. , Kuhn, H. , & Aigner, L. (2005). Doublecortin expression levels in adult brain reflect neurogenesis. European Journal of Neuroscience, 21(1), 1–14. 10.1111/j.1460-9568.2004.03813.x 15654838

[brb32847-bib-0009] Dai, X. , Lu, X. , Cheng, F. , Hao, H. , Qian, T. , Yu, W. , Tang, L. , & Li, L. (2015). Neurogenin 2 enhances the neuronal differentiation of skin‐derived precursors. International Journal of Neuroscience, 125(5), 367–374. 10.3109/00207454.2014.935375 24946204

[brb32847-bib-0010] Dayer, A. G. , Ford, A. A. , Cleaver, K. M. , Yassaee, M. , & Cameron, H. A. (2003). Short‐term and long‐term survival of new neurons in the rat dentate gyrus. Journal of Comparative Neurology, 460(4), 563–572. 10.1002/cne.10675 12717714

[brb32847-bib-0011] Deng, B. , Gou, X. , Chen, H. , Li, L. , Zhong, H. , Xu, H. , Jiang, F. , Zhao, Z. , Wang, Q. , & Xu, L. (2013). Targeted delivery of neurogenin‐2 protein in the treatment for cerebral ischemia‐reperfusion injury. Biomaterials, 34(34), 8786–8797. 10.1016/j biomaterials.2013.07.076 23942209

[brb32847-bib-0012] Dong, Y. , Li, S. , Lu, Y. , Li, X. , Liao, Y. , Peng, Z. , Li, Y. , Hou, L. , Yuan, Z. , & Cheng, J. (2020). Stress‐induced NLRP3 inflammasome activation negatively regulates fear memory in mice. Journal of Neuroinflammation, 17(1), 205. 10.1186/s12974-020-01842-0 32635937 PMC7341659

[brb32847-bib-0013] Feigin, V. L. , Krishnamurthi, R. V. , Parmar, P. , Norrving, B. , Mensah, G. A. , Bennett, D. A. , Barker‐Collo, S. , Moran, A. E. , Sacco, R. L. , Truelsen, T. , Davis, S. , Pandian, J. D. , Naghavi, M. , Forouzanfar, M. H. , Nguyen, G. , Johnson, C. O. , Vos, T. , Meretoja, A. , Murray, C. J. L. , & Roth, G. A. (2015). Update on the global burden of ischemic and hemorrhagic stroke in 1990–2013: The GBD 2013 Study. Neuroepidemiology, 5(3), 161–176. 10.1159/000441085 PMC4633282 26505981

[brb32847-bib-0014] Fuss, J. , Ben Abdallah, N. M. , Vogt, M. A. , Touma, C. , Pacifici, P. G. , Palme, R. , Witzemann, V. , Hellweg, R. , & Gass, P. (2010). Voluntary exercise induces anxiety‐like behavior in adult C57BL/6J mice correlating with hippocampal neurogenesis. Hippocampus, 20(3), 364–376. 10.1002/hipo.20634 19452518

[brb32847-bib-0015] Hand, R. , & Polleux, F. (2011). Neurogenin2 regulates the initial axon guidance of cortical pyramidal neurons projecting medially to the corpus callosum. Neural Development, 6, 30. 10.1186/1749-8104-6-30 21864333 PMC3174110

[brb32847-bib-0016] Heng, J. I. , Nguyen, L. , Castro, D. S. , Zimmer, C. , Wildner, H. , Armant, O. , Skowronska‐Krawczyk, D. , Bedogni, F. , Matter, J. , Hevner, R. , & Guillemot, F. (2008). Neurogenin 2 controls cortical neuron migration through regulation of Rnd2. Nature, 455(7209), 114–118. 10.1038/nature0719 18690213

[brb32847-bib-0017] Ho, S. M. , Hartley, B. J. , Tcw, J. , Beaumont, M. , Stafford, K. , Slesinger, P. A. , & Brennand, K. J. (2016). Rapid Ngn2‐induction of excitatory neurons from hiPSC‐derived neural progenitor cells. Methods (San Diego, Calif.), 101, 113–124. 10.1016/j.ymeth.2015.11.019 26626326 PMC4860098

[brb32847-bib-0018] Ishikawa, M. , Aoyama, T. , Shibata, S. , Sone, T. , Miyoshi, H. , Watanabe, H. , Nakamura, M. , Morota, S. , Uchino, H. , Yoo, A. S. , & Okano, H. (2020). miRNA‐based rapid differentiation of purified neurons from hPSCs advances towards quick screening for neuronal disease phenotypes in vitro. Cells, 9(3), 532. 10.3390/cells9030532 32106535 PMC7140514

[brb32847-bib-0019] Kawai, T. , Takagi, N. , Miyake‐Takagi, K. , Okuyama, N. , Mochizuki, N. , & Takeo, S. (2004). Characterization of BrdU‐positive neurons induced by transient global ischemia in adult hippocampus. Journal of Cerebral Blood Flow and Metabolism, 24(5), 548–555. 10.1097/00004647-200405000-00009 15129187

[brb32847-bib-0020] Kitagawa, K. , Matsumoto, M. , Yang, G. , Mabuchi, T. , Yagita, Y. , Hori, M. , & Yanagihara, T. (1998). Cerebral ischemia after bilateral carotid artery occlusion and intraluminal suture occlusion in mice: Evaluation of the patency of the posterior communicating artery. Journal of Cerebral Blood Flow and Metabolism, 18(5), 570–579. 10.1097/00004647-199805000-00012 9591849

[brb32847-bib-0021] Koh, S. H. , & Park, H. H. (2017). Neurogenesis in stroke recovery. Translational Stroke Research, 8(1), 3–13. 10.1007/s12975-016-0460-z 26987852

[brb32847-bib-0022] Kuhn, H. G. (2015). Control of cell survival in adult mammalian neurogenesis. Cold Spring Harbor Perspectives in Biology, 7(12), a018895. 10.1101/cshperspect.a018895 26511628 PMC4665071

[brb32847-bib-0023] Kurozumi, K. , Nakamura, K. , Tamiya, T. , Kawano, Y. , Kobune, M. , Hirai, S. , Uchida, H. , Sasaki, K. , Ito, Y. , Kato, K. , Honmou, O. , Houkin, K. , Date, I. , & Hamada, H. (2004). BDNF gene‐modified mesenchymal stem cells promote functional recovery and reduce infarct size in the rat middle cerebral artery occlusion model. Molecular Therapy, 9(2), 189–197. 10.1016/j.ymthe.2003.10.012 14759803

[brb32847-bib-0024] León‐Moreno, L. C. , Castañeda‐Arellano, R. , Rivas‐Carrillo, J. D. , & Dueñas‐Jiménez, S. H. (2020). Challenges and improvements of developing an ischemia mouse model through bilateral common carotid artery occlusion. Journal of Stroke & Cerebrovascular Diseases, 29(5), 104773. 10.1016/j.jstrokecerebrovasdis.2020.104773 32199775

[brb32847-bib-0025] Maysami, S. , Lan, J. Q. , Minami, M. , & Simon, R. P. (2008). Proliferating progenitor cells: A required cellular element for induction of ischemic tolerance in the brain. Journal of Cerebral Blood Flow and Metabolism, 28(6), 1104–1113. 10.1038/jcbfm.2008.4 18319730 PMC5997187

[brb32847-bib-0026] Milot, M. R. , & Plamondon, H. (2009). Time‐dependent effects of global cerebral ischemia on anxiety, locomotion, and habituation in rats. Behavioural Brain Research, 200(1), 173–180. 10.1016/j.bbr.2009.01.009 19378462

[brb32847-bib-0027] Moreno‐Jimenez, E. P. , Flor‐Garcia, M. , Terreros‐Roncal, J. , Rábano, A. , Cafini, F. , Pallas‐Bazarra, N. , Ávila, J. , & Llorens‐Martín, M. (2019). Adult hippocampal neurogenesis is abundant in neurologically healthy subjects and drops sharply in patients with Alzheimer's disease. Nature Medicine, 25(4), 554–560. 10.1038/s41591-019-0375-9 30911133

[brb32847-bib-0028] Pforte, C. , Henrich‐Noack, P. , Baldauf, K. , & Reymann, K. G. (2005). Increase in proliferation and gliogenesis but decrease of early neurogenesis in the rat forebrain shortly after transient global ischemia. Neuroscience, 136(4), 1133–1146. 10.1016/j.neuroscience.2005.08.043 16216427

[brb32847-bib-0029] Richetin, K. , Steullet, P. , Pachoud, M. , Perbet, R. , Parietti, E. , Maheswaran, M. , Eddarkaoui, S. , Bégard, S. , Pythoud, C. , Rey, M. , Caillierez, R. , Q Do, K. , Halliez, S. , Bezzi, P. , Buée, L. , Leuba, G. , Colin, M. , Toni, N. , & Déglon, N. (2020). Tau accumulation in astrocytes of the dentate gyrus induces neuronal dysfunction and memory deficits in Alzheimer's disease. Nature Neuroscience, 23(12), 1567–1579. 10.1038/s41593-020-00728-x 33169029

[brb32847-bib-0030] Roll, L. , & Faissner, A. (2014). Influence of the extracellular matrix on endogenous and transplanted stem cells after brain damage. Front Cell Neuroscience, 8, 219. 10.3389/fncel.2014.00219 PMC4137450 25191223

[brb32847-bib-0031] Rossi, C. , Angelucci, A. , Costantin, L. , Braschi, C. , Mazzantini, M. , Babbini, F. , Fabbri, M. E. , Tessarollo, L. , Maffei, L. , Berardi, N. , & Caleo, M. (2006). Brain‐derived neurotrophic factor (BDNF) is required for the enhancement of hippocampal neurogenesis following environmental enrichment. European Journal of Neuroscience, 24(7), 1850–1856. 10.1111/j.1460-9568.2006.05059.x 17040481

[brb32847-bib-0032] Schörnig, M. , Ju, X. , Fast, L. , Ebert, S. , Weigert, A. , Kanton, S. , Schaffer, T. , NadifKasri, N. , Treutlein, B. , Peter, B. M. , Hevers, W. , & Taverna, E. (2021). Comparison of induced neurons reveals slower structural and functional maturation in humans than in apes. Front Cell Neuroscience, 10, e59323. 10.7554/eLife.59323.sa2 PMC7870144 33470930

[brb32847-bib-0033] Serre, A. , Snyder, E. Y. , Mallet, J. , & Buchet, D. (2012). Overexpression of basic helix‐loop‐helix transcription factors enhances neuronal differentiation of fetal human neural progenitor cells in various ways. Stem Cells and Development, 21(4), 539–553. 10.1089/scd.2011.0079 21561385 PMC3280607

[brb32847-bib-0034] Shen, J. , Bai, X. Y. , Qin, Y. , Jin, W. W. , Zhou, J. Y. , Zhou, J. P. , Yan, Y. G. , Wang, Q. , Bruce, I. C. , Chen, J. H. , & Xia, Q. (2011). Interrupted reperfusion reduces the activation of NADPH oxidase after cerebral I/R injury. Free Radical Biology and Medicine, 50(12), 1780–1786. 10.1016/j.freeradbiomed.2011.03.028 21458562

[brb32847-bib-0035] Silva, R. , Abrunheiro, S. , Cardoso, D. , Costa, P. , Couto, F. , Agrenha, C. , & Apóstolo, J. (2018). Effectiveness of multisensory stimulation in managing neuropsychiatric symptoms in older adults with major neurocognitive disorder: a systematic review. JBI Database of Systematic Reviews and Implementation Report, 16(8), 1663–1708. 10.11124/JBISRIR-2017-003483 30113550

[brb32847-bib-0036] Soares, L. M. , Schiavon, A. P. , Milani, H. , & de Oliveira, R. M. (2013). Cognitive impairment and persistent anxiety‐related responses following bilateral common carotid artery occlusion in mice. Behavioural Brain Research, 249, 28–37. 10.1016/j.bbr.2013.04.010 23602921

[brb32847-bib-0037] Sun, J. , Li, H. , Jin, Y. , Yu, J. , Mao, S. , Su, K. P. , Ling, Z. , & Liu, J. (2021). Probiotic Clostridium butyricum ameliorated motor deficits in a mouse model of Parkinson's disease via gut microbiota‐GLP‐1 pathway. Brain, Behavior, and Immunity, 91, 703–715. 10.1016/j.bbi.2020.10.014 33148438

[brb32847-bib-0038] Tang, L. , Lu, X. , Zhu, R. , Qian, T. , Tao, Y. , Li, K. , Zheng, J. , Zhao, P. , Li, S. , Wang, X. , & Li, L. (2016). Adipose‐derived stem cells expressing the neurogenin‐2 promote functional recovery after spinal cord injury in rat. Cellular and Molecular Neurobiology, 36(5), 657–667. 10.1007/s10571-015-0246-y 26283493 PMC11482400

[brb32847-bib-0039] Thoma, E. C. , Wischmeyer, E. , Offen, N. , Maurus, K. , Sirén, A. L. , Schartl, M. , & Wagner, T. U. (2012). Ectopic expression of neurogenin 2 alone is sufficient to induce differentiation of embryonic stem cells into mature neurons. PLoS One, 7(6), e38651. 10.1371/journal.pone.0038651 22719915 PMC3374837

[brb32847-bib-0040] Yang, Z. R. , Wang, H. Y. Y. , Edwards, D. , Ding, C. , Yan, L. , Brayne, C. , & Mant, J. (2020). Association of blood lipids, atherosclerosis and statin use with dementia and cognitive impairment after stroke: A systematic review and meta‐analysis. Ageing Research Reviews, 57, 100962. 10.1016/j.arr.2019.100962 31505259

[brb32847-bib-0041] Yoshioka, H. , Niizuma, K. , Katsu, M. , Sakata, H. , Okami, N. , & Chan, P. H. (2011). Consistent injury to medium spiny neurons and white matter in the mouse striatum after prolonged transient global cerebral ischemia. Journal of Neurotrauma, 28(4), 649–660. 10.1089/neu.2010 1662, 21309724 PMC3070149

[brb32847-bib-0042] Zhao, Y. , Wang, J. , Du, J. , Li, B. , Gou, X. , Liu, J. , Hou, L. , Sang, H. , & Deng, B. (2018). TAT‐Ngn2 enhances cognitive function recovery and regulates caspase‐dependent and mitochondrial apoptotic pathways after experimental stroke. Frontiers in Cell Neuroscience, 12, 475. 10.3389/fncel.2018.00475 PMC6302814 30618628

